# A percutaneous biliary anastomotic reconstruction for a failed Roux-en-Y hepaticojejunostomy in a patient with cholangiocarcinoma: A case report

**DOI:** 10.1016/j.radcr.2022.09.013

**Published:** 2022-10-03

**Authors:** Zachary Cohen, Shimon Aronhime

**Affiliations:** a Hackensack Health Ocean University Medical Center, Brick, NJ, USA; b Department of Interventional Radiology, Shamir Medical Center, Israel

## Abstract

We hereby report a case of a novel percutaneous biliary anastomotic reconstruction of a disconnected segment 2 bile duct to the roux limb in a patient with cholangiocarcinoma suffering from bile leak post trisegmentectomy and roux-en-y hepaticojejunostomy. The patient was not a candidate for surgical reanastomosis and was suffering from repeated episodes of cholangitis prior to our intervention. We were successfully able to resolve the patient's biliary symptoms and need for an external biliary collection bag using our technique. The patient's case and the details of our intervention with relevant imaging is discussed. A review on the management of biliary leak following roux-en-y hepaticojejunostomy is also included.

## Introduction

Cholangiocarcinoma is a relatively rare type of epithelial cancer that is characterized by late diagnosis and poor outcome [1]. Although there is no definite cure for cholangiocarcinoma, surgical resection of the affected hepatobiliary system remains the mainstay of treatment for patients deemed good surgical candidates. For such patients, a Roux-en-Y hepaticojejunostomy (HJ) is commonly performed to reconstruct flow of bile to the GI tract [2]. Recognized complications of Roux-en-Y HJ include postoperative anastomotic stricture formation or complete anastomotic failure [3,4]. Treatment options for anastomotic strictures generally follow a percutaneous, endoscopic or surgical approach. Conversely, anastomotic failures are usually treated via surgical reoperation due to better outcomes and poor percutaneous or endoscopic reconstructive alternatives. We hereby report a case of a percutaneous biliary anastomotic reconstruction of a disconnected segment 2 bile duct to the roux limb in a patient who was not a candidate for open bile duct reconstructive surgery.

## Case description

A 64-year-old man was diagnosed with cholangiocarcinoma that was extending past the bifurcation of the bile ducts and adjacent to the origin of the left portal vein, with associated atrophy of the right liver lobe, hypertrophy of the left liver lobe and caudate, and no distant metastases. An internal-external biliary drainage catheter was placed for biliary obstruction (total bilirubin = 2.95 mg/dL) and chemotherapy was initiated with Cisplatin and Gemcitabine. The patient demonstrated good clinical and imaging responses over the course of a year, and subsequently underwent hepatic trisegmentectomy with a Roux-en-Y HJ. Unfortunately, the anastomosis of the segment 2 bile duct to the roux-limb was not successful and a biloma developed postoperatively. A biliary drainage catheter was placed through the segment 2 duct into the biloma, yet the patient continued to suffer from recurrent bouts of cholangitis. The patient was then taken to surgery in an attempt to reconstruct the segment 2 bile duct but was deemed inoperable due to very difficult adhesions and increased risk of injury to the intestine and segment 3 bile duct. Expectedly, the patient continued to present with repeated biliary symptoms that were treated with antibiotics and biliary catheter exchanges.

**Fig. 1:**
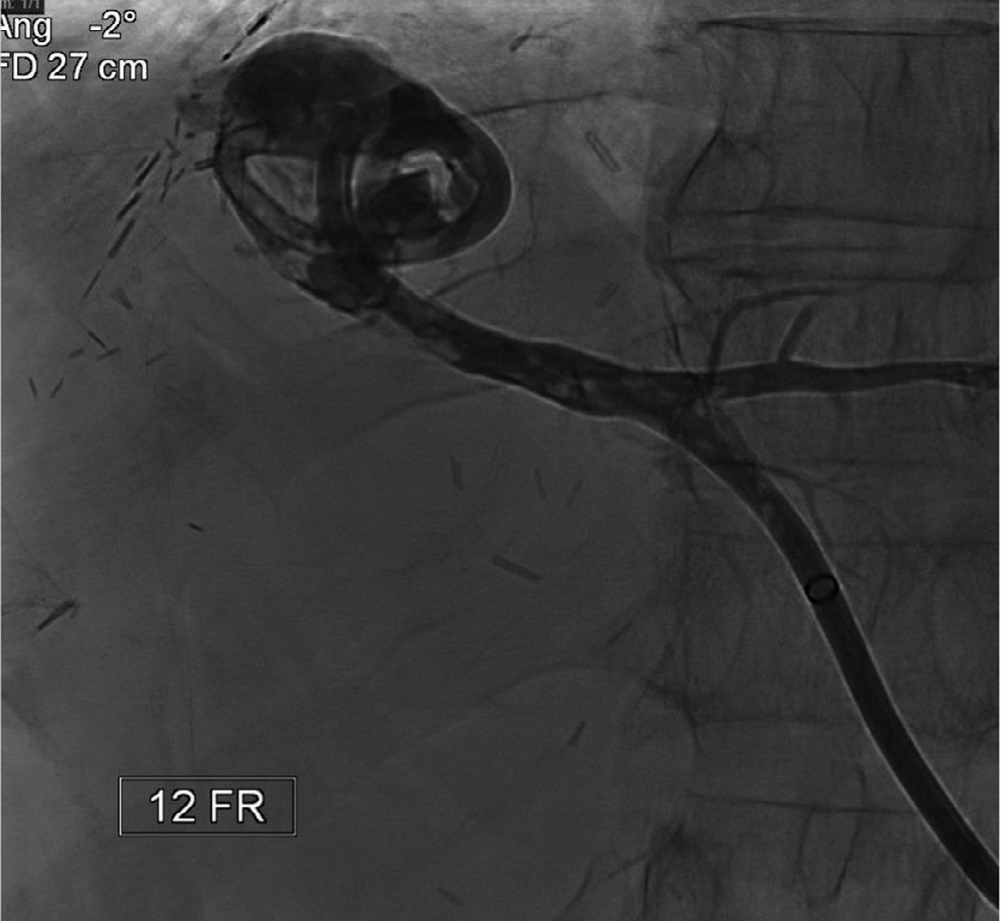
Preoperative imaging demonstrating a segment 2 biliary biloma with indwelling drain.

**Fig. 2:**
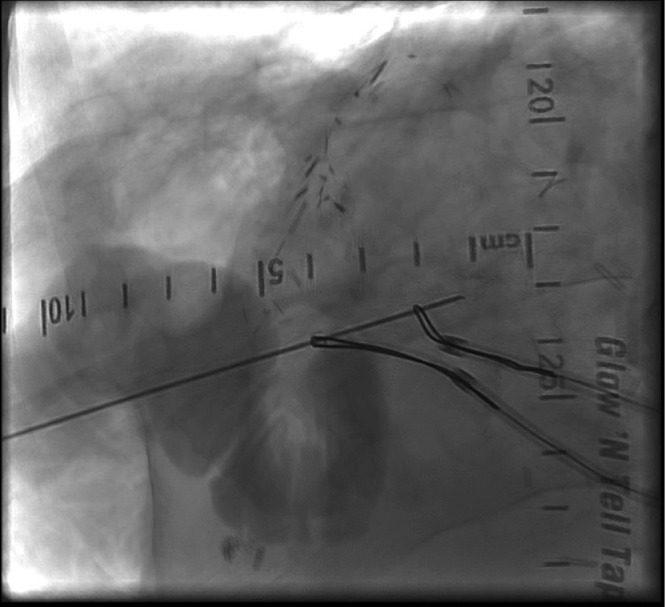
Snares are placed within the segment 2 biloma and small bowel (via segment 3). Using the gunsight technique, a percutaneous needle is placed through both snares.

**Fig. 3:**
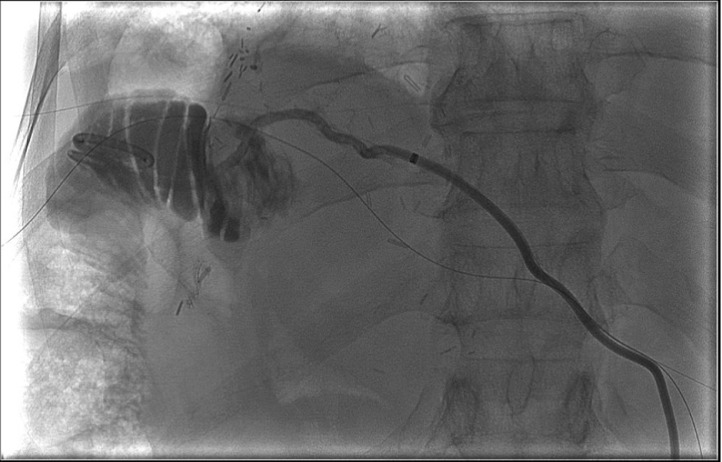
Biliary anastomotic reconstruction with internal external drainage of the segment 2 duct with pigtail in the small bowel.

The patient was in need of an alternative treatment plan and was admitted to our IR service for a percutaneous biliary reconstruction of their disconnected segment 2 duct to the roux limb. The procedure was performed under general anesthesia. Initial imaging again demonstrated the segment 2 bile duct terminating in a biloma (Fig. 1). The segment 2 drainage catheter was exchanged for a sheath and a snare was placed into the biloma. Access was then obtained to the segment 3 duct. A sheath was then placed, and another snare was placed across the normal anastomosis into the small bowel. Using the gunsight technique, a long 20-g needle was then percutaneously advanced through both snares (Fig. 2). Cone beam CT was used to confirm adequate placement of the needle. The needle was exchanged for a wire, and a 10 fr internal-external biliary drainage catheter was placed from the segment 2 bile duct across the biloma and into the small bowel (Fig. 3).

At 3 months postoperative follow-up, the drain was upsized from a 10 F to 12 F catheter and the drain was capped. At 6 months postoperative follow-up, the patient was doing well and denied any biliary symptoms. The catheter was then exchanged for a safety Kumpe that remains in place. The patient is symptom free with current deliberations ongoing as to whether and when to remove the safety Kumpe catheter.

## Discussion

Bile leak is defined as bilious secretion with more than 3 times the serum concentration of bilirubin on or after day 3, or in our case, the necessity of intervention due to collections of biliary fluid [5]. Moreover, the incidence of biliary leakage following biliary-enteric anastomoses is 2%-10% [6]. Anastomotic failure with resultant biliary leakage can present with episodes of cholangitis, biloma formation, sepsis, and multiorgan failure. When investigating a potential anastomotic biliary leak, it is important to verify the exact site of biliary leakage via radiographic means. Other potential sites of bile leak include the hepatic parenchymal resection margin in the case of liver resection, the closed end of the jejunal loop, and injuries to other bile ducts outside of the anastomotic regions [6]. Our leak was verified to be from the failed segment 2 anastomosis to the roux limb.

Definitive treatment typically requires biliary drainage followed by biliary reconstructive surgery. For nonsurgical candidates, percutaneous interventions may treat the leak if the catheter is able to cross from the bile duct into the small bowel and provide a scaffold for healing. Percutaneous transhepatic cholangiography followed by percutaneous transhepatic biliary drainage (PTBD) is the most common percutaneous treatment option for bile leak. Success rates of PTBD with an internal-external catheter for biliary leakage are reported to be 50%-100% without secondary surgery [7]. However, the technique does have certain drawbacks. For example, it is unclear if the catheter should be removed when cholangiography confirms resolution of the leak without stenosis, or if it should be left in-situ for longer. Hence, the average time range from PTBD to closure of the leak is reported as 9-150 days. Moreover, secondary complications such as hemorrhage, residual biliary stenosis, ductal perforation, and metabolic acidosis from continuous bile loss have been reported [7]. One must also not overlook the physical and psychosocial burden on the patient having to live life with an external collection bag. Thus, for patients who are not surgical candidates and for whom biliary drainage is not curative, treatment options for biliary anastomotic failures are very limited.

Our patient was not deemed a candidate for surgery and therefore not able to receive surgical re-anastomosis. Moreover, his bile leak was refractory to external-internal biliary drainage causing him to suffer from repeat hospitalizations due to cholangitis. The patient was in need of a nonsurgical alternative treatment plan. We were able to resolve the patient's repeated bouts of cholangitis and eliminate the need for an external collection bag via our percutaneous anastomotic reconstruction. Follow-up with our patient 6 months post-op revealed patent anastomoses with good flow, normal bilirubin levels, and no reported symptoms.

## Conclusion

Our percutaneous biliary anastomotic reconstruction afforded an alternative therapeutic approach to solving anastomotic failure in a patient who was not a surgical candidate and nonresponsive to internal-external biliary drainage. This allowed our patient to achieve a better quality of life by reducing hospitalizations and eliminating the need for an external biliary collection bag. The success of our intervention in this case warrants further studies to compare and contrast its long-term effectiveness to that of surgical retreatment and standard PTBD approaches for anastomotic failures.

## Ethical approval

All procedures performed in studies involving human participants were in accordance with the ethical standards of the institutional and/or national research committee and with the 1964 Helsinki declaration and its later amendments or comparable ethical standards.

## Patient consent

Written informed consent was obtained from all individual participants included in the study.
